# A Two-Hour Fetal Glucagon Infusion Stimulates Hepatic Catabolism of Amino Acids in Fetal Sheep

**DOI:** 10.3390/ijms26051904

**Published:** 2025-02-22

**Authors:** Amelia R. Tanner, Sarah N. Cilvik, Marjorie A. Nguyen, Evgenia Dobrinskikh, Russell V. Anthony, Stephanie R. Wesolowski, Paul J. Rozance

**Affiliations:** 1 Perinatal Research Center, Department of Pediatrics, University of Colorado School of Medicine, Aurora, CO 80045, USA; 2 Department of Pediatrics, Wake Forest University School of Medicine, Winston-Salem, NC 27101, USA; 3 Department of Biomedical Sciences, Colorado State University, Fort Collins, CO 80523, USA

**Keywords:** glucagon, fetus, liver, amino acids, gluconeogenesis

## Abstract

Postnatally, glucagon acutely lowers plasma amino acid (AA) concentrations by stimulating hepatic AA catabolism, but its fetal actions remain unclear. This study tested whether a 2 h fetal glucagon infusion would stimulate hepatic AA catabolism and inhibit placental AA transfer. Late-gestation pregnant sheep (0.9 gestation) underwent surgical, vascular catheterization and received fetal glucagon (*n* = 8) or vehicle infusions (*n* = 8) in a crossover design with a 48 h washout period. Nutrient uptake and utilization were assessed during each infusion, and fetal liver and placental tissue were collected post-infusion under hyperglucagonemic (*n* = 4) or vehicle (*n* = 4) conditions. Glucagon receptor was identified in fetal hepatocyte and trophoblast cells. Glucagon reduced fetal plasma AA concentrations by 20% (*p* = 0.0103) and increased plasma glucose by 47% (*p* = 0.0152), leading to a three-fold rise in fetal plasma insulin (*p* = 0.0459). Hepatic gene expression associated with AA catabolism and gluconeogenesis increased (*p* < 0.0500) following glucagon infusion, and hepatic metabolomic analysis showed enrichment in AA metabolism pathways. However, placental AA transfer was unaffected by 2 h fetal glucagon infusions. In conclusion, a 2 h glucagon infusion stimulates hepatic glucose production and enhances AA catabolism in the fetal liver, lowering plasma AA concentrations. The primary acute effects of fetal glucagon are hepatic, as placental AA transfer is unchanged.

## 1. Introduction

Glucagon is secreted by the pancreatic α-cells in response to fasting or hypoglycemia [1]. This secretion elicits tissue-specific glucoregulatory responses, including the activation of glycogenolysis and gluconeogenesis in the liver [1]. Glucagon has also been shown to stimulate amino acid catabolism and ureagenesis in the liver [2]. The regulatory relationship between amino acid (AA) metabolism and glucagon in the adult has been recognized for over 30 years and involves an AA-glucagon feedback loop [3,4,5,6]. In response to increased circulating AAs, glucagon is released by the pancreas, which, in turn, stimulates hepatic AA catabolism and lowers AA concentrations [6,7]. This mechanism establishes glucagon as a central hormone in a homeostatic feedback loop to maintain circulating AAs in a physiological concentration range in the adult [6,7].

The role of glucagon in regulating fetal metabolism, however, is less well characterized. This knowledge gap has important clinical ramifications, as elevated fetal glucagon concentrations have been observed in response to intrauterine acidosis, hypoxia, and growth restriction [8,9]. Fetal glucagon concentrations are also increased in experiment sheep models of placental insufficiency-induced fetal growth restriction (FGR), maternal starvation, hypoxia, and elevated catecholamines [10,11,12,13,14]. However, until recently, little was known about the physiological significance of elevated fetal glucagon as a driver of fetal nutrient metabolism rather than simply as a marker of fetal distress. In fact, it is possible that glucagon also plays a role in AA homeostasis in adults, but this relationship may be more complex as the fetal AA requirements are supplied by the placenta. Our previous studies support this idea, as fetal glucagon infusion for 8–10 d lowered fetal AA concentrations through inhibition of placental transport of AAs into fetal circulation [15].

To more directly assess the acute impact of glucagon on the fetal liver and in the regulation of fetal AA concentrations, two-hour glucagon infusions were selected. Our hypothesis was that two-hour fetal glucagon infusions would lower fetal AA concentrations by both acutely stimulating hepatic AA catabolism and reducing placental AA transfer. To test this, we examined the steady-state flux of AAs across the placenta under both baseline and hyperglucagonemic conditions and by collecting fetal hepatic tissue under the same conditions to determine the capacity for glucagon to acutely stimulate regulators of AA catabolism and gluconeogenesis.

## 2. Results

### 2.1. Nutrient and Hormone Concentrations

**Figure 1:**
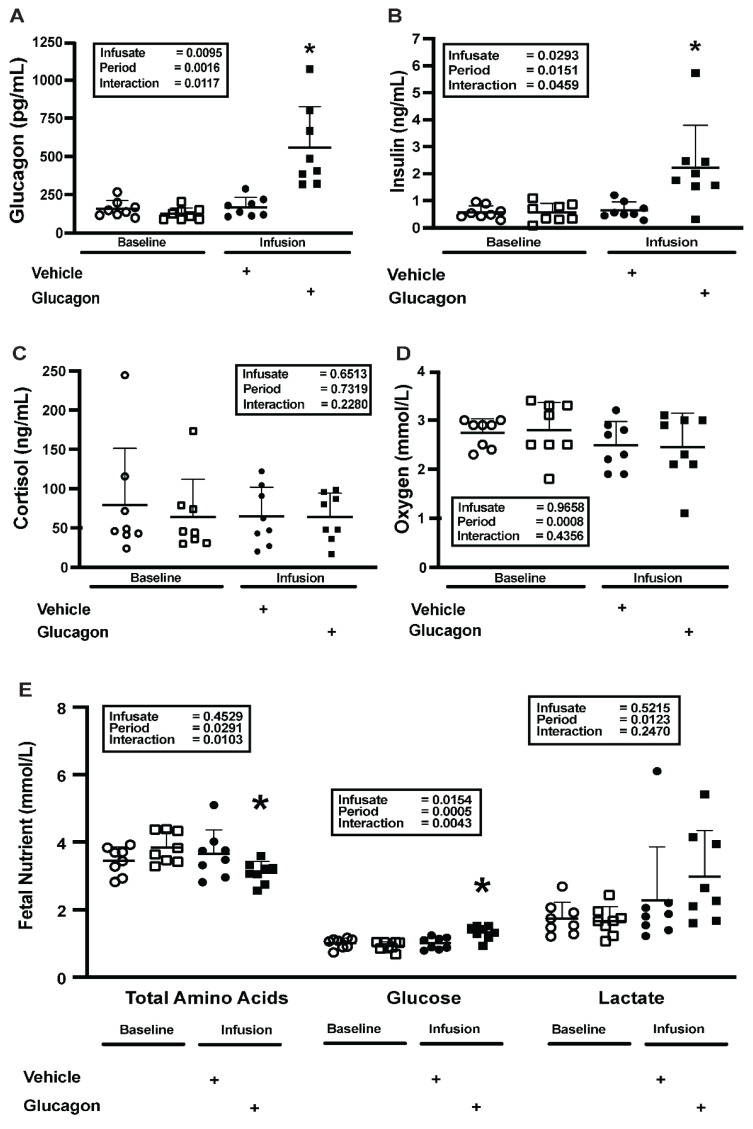
Two-hour fetal glucagon infusions lower AA and increase glucose. (**A**) Fetal plasma glucagon, (**B**) insulin, and (**C**) cortisol concentrations, as well as (**D**) fetal blood oxygen concentrations, and (**E**) fetal plasma AA (total), glucose, and lactate concentrations at baseline (open symbols) and in response to a two-hour vehicle (closed circles) or glucagon (closed squares) infusion. Results were compared with a mixed models ANOVA that included a random animal term to account for repeated measurements within the same animal. *p*-Values or the main effects of infusate (vehicle vs. glucagon), period (baseline vs. infusion), and their interaction are in the inset. * indicates a significant difference (*p* < 0.05) between glucagon infusion (*n* = 4) vs. vehicle infusion (*n* = 4) and baseline (*n* = 8). Individual values, means, and +SDs are shown.

During the fetal glucagon infusion, fetal arterial plasma glucagon concentrations increased 350% over baseline (*p* = 0.0117, Figure 1A) but were unchanged by vehicle infusion. By the end of the fetal glucagon infusion, fetal arterial plasma insulin concentrations had increased three-fold over baseline (*p* = 0.0459, Figure 1B) but did not increase during the vehicle infusion. Fetal arterial plasma cortisol concentrations did not change as a result of either infusion (Figure 1C). As 9 d fetal glucagon infusions dramatically lowered maternal chorionic somatomammotropin (CSH), we measured CSH concentrations and placental secretion in the current study. Neither uterine vein CSH concentrations nor uteroplacental CSH secretion were impacted by glucagon infusion (Table 1).

To assess the acute actions of glucagon on nutrient homeostasis, fetal nutrient concentrations were analyzed. Fetal (Figure 1D) blood oxygen concentrations did not change during either the glucagon or vehicle infusion. Fetal arterial plasma summed AA concentrations (sum total) decreased by 20% (*p* = 0.0103; Figure 1E), and glucose concentrations increased by 47% (*p* = 0.0043; Figure 1E) during the glucagon infusion. Neither glucose nor AA concentrations changed during the vehicle infusion. Fetal arterial lactate concentrations increased during both the glucagon and vehicle infusions (*p* = 0.0123; Figure 1E), but there were no differences between the two infusions.

Prior to either infusion, higher fetal glucagon concentrations were associated (*p* = 0.0171) with lower total fetal plasma AA concentrations as described by the equation Y = −0.005615∗X + 4.431 (Supplemental Figure S1). Fetal arterial plasma concentrations of individual AAs are shown in Supplemental Table S2. Fetal arterial AA concentrations decreased (*p* < 0.0500) during the glucagon infusion for all essential AAs except for methionine. The same pattern was observed (*p* < 0.0500) for six non-essential AAs and ornithine. Maternal oxygen summed AA (sum total), glucose, and lactate arterial plasma concentrations did not change during either the glucagon or vehicle infusion compared with baseline (Table 1).

### 2.2. Blood Flow and Nutrient Uptake Rates

To determine if lower fetal AA concentrations were driven by changes in placental transport, we looked at nutrient uptake rates. Umbilical blood flow rates and uptake rates of total AA carbon, total AA nitrogen, lactate, total nutrient carbon, and oxygen did not change during either the glucagon or vehicle infusion (Table 1). Umbilical glucose uptake rates decreased by 36% during the glucagon infusion (*p* = 0.0152, Table 1) but did not change during the vehicle infusion. Specific umbilical AA uptake rates are shown in Supplemental Table S3. There were no statistically significant changes for any of the specific umbilical AA uptake rates except for arginine and asparagine, which both increased (*p* < 0.0500) slightly during the vehicle infusion and decreased (*p* < 0.0500) slightly during the glucagon infusion.

Uterine blood flow rates and uptake rates of total AA carbon, total AA nitrogen, glucose, lactate, total nutrient carbon, and oxygen did not change during the glucagon or vehicle infusion (Table 1). Individual uterine AA uptake rates did not change during either infusion (Supplemental Table S4). Uteroplacental utilization rates for glucose, lactate, and oxygen did not change during either infusion (Table 1).

### 2.3. Glucagon Receptor Localization, Relative mRNA, and Protein Concentrations

**Figure 2:**
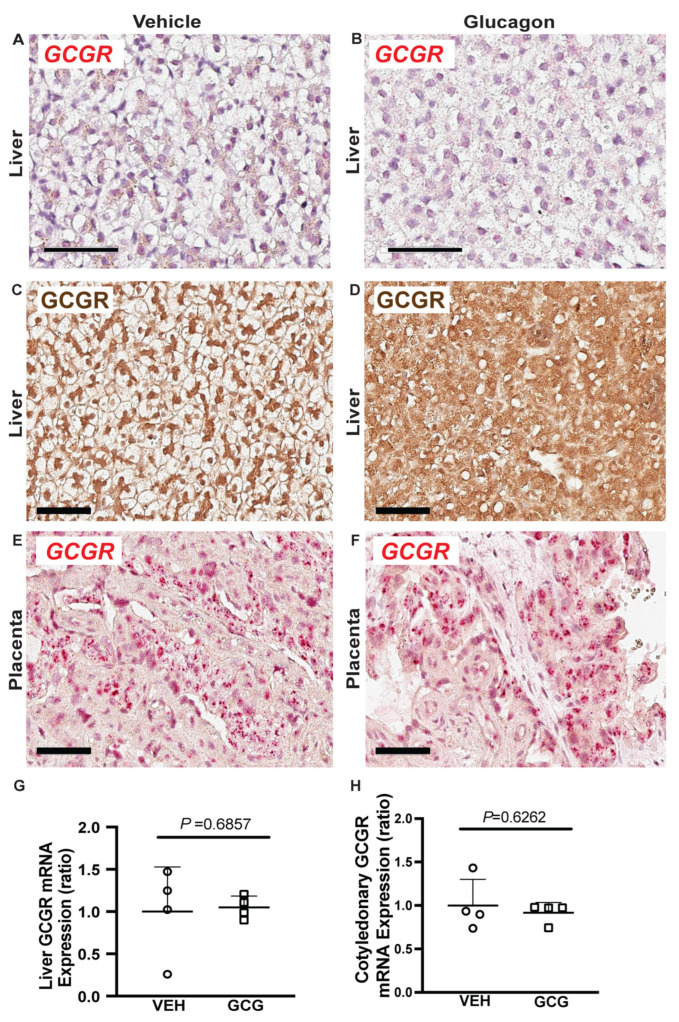
Glucagon receptor message and protein are present in the fetal liver and trophoblast of the sheep placenta, but mRNA concentrations do not change with two-hour fetal glucagon infusions. Fetal liver glucagon receptor mRNA (red) is present in the fetal liver as evidenced by RNAscope in both (**A**) control and (**B**) fetal glucagon-exposed fetal livers. (**C**,**D**) Fetal liver glucagon receptor protein (brown) is primarily located on the plasma membrane and within the nucleus of vehicle (VEH) infused fetal livers. However, in tissue collected under hyperglucagonemic conditions (GCG), greater concentrations of GCGR within the cytoplasm are detected. Placental glucagon receptor message (RNAscope, red) is present within the cytoplasm of the trophoblast of the sheep placenta in both (**E**) control and (**F**) fetal glucagon-exposed placenta. The scale bar is 60 μm. (**G**) Fetal liver GCGR mRNA concentrations normalized to the average of two reference genes. The results of the tissue collected under hyperglucagonemic conditions (GCG) are presented as fold change relative to vehicle infused group (VEH). (**H**) Cotyledonary mRNA concentrations of the glucagon receptor (GCGR) were normalized to the average of two reference genes. The results of the tissue collected under hyperglucagonemic conditions (GCG) are presented as fold change relative to vehicle group (VEH). Results were compared by the Mann-Whitney test. Individual values, means, +SDs, and *p*-Values are shown.

To further explore the mechanism behind lower fetal AA concentrations, we examined GCGR localization and gene expression in the liver. GCGR mRNA (red) localizes in situ to all hepatocytes and was not impacted by two-hour glucagon exposure (Figure 2A,B). Under vehicle infusion conditions, GCGR protein localizes primarily near the nucleus and the cell periphery (Figure 2C). However, in response to two hours of glucagon infusion, GCGR signal distribution shifts, more evenly covering the cytoplasm with less obvious cell boundaries, which could possibly be due to internalization (Figure 2D).

Placental glucagon receptor mRNA (red; Figure 2E,F) localizes in situ primarily to the cotyledonary tissue (fetal) of the placentome, mainly within the trophoblast and is not impacted by glucagon infusion. Neither fetal liver (Figure 2G) nor placental tissue relative mRNA concentrations (Figure 2H) or protein concentrations (Supplemental Figure S2) of GCGR differed between tissues collected under glucagon or vehicle infusion conditions. Additionally, no differences in placental relative mRNA concentrations of CSH were observed between placentas collected under either infusion condition (Supplemental Figure S3).

### 2.4. Metabolic and Metabolomic Characteristics of the Fetal Liver

**Figure 3:**
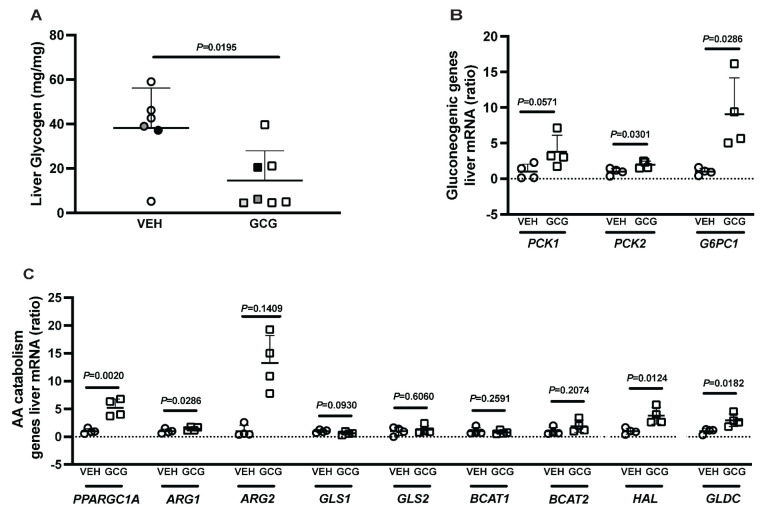
Two-hour fetal glucagon infusion increases mRNA concentrations of several genes related to glucose and amino acid metabolism and lowers the glycogen content of the fetal liver. (**A**) Fetal liver glycogen content was lower in tissue collected under hyperglucagonemic conditions (GCG) compared to vehicle infusion group conditions or a non-infused state (VEH). Symbols filled in black or gray represent twins from the same pregnancy. (**B**) Fetal liver mRNA concentrations of glucogeneogenic genes and (**C**) amino acid catabolizing genes were normalized to the average of two reference genes. The results of the tissue collected under hyperglucagonemic conditions (GCG) are presented as fold change relative to the vehicle infusion group (VEH). Results were compared using the Mann–Whitney test. Individual values, means, +SDs, and *p*-Values are shown.

Due to the decrease in circulating fetal AA concentrations and the redistribution of GCGR in the fetal liver, we wanted to understand the hepatic transcriptomic and metabolomic changes in response to 2 h fetal glucagon infusions. In the fetal liver, glycogen content was 62% lower in the animals sacrificed under glucagon infusion conditions compared with vehicle infusion (*p* = 0.0195, Figure 3A). Furthermore, glucagon increased relative fetal hepatic mRNA concentrations of the gluconeogenic genes PCK2 (97% higher, *p* = 0.0301; Figure 3B) and G6PC1 (807% higher, *p* = 0.0286; Figure 3B). PCK1, a key gluconeogenic enzyme, was 278% higher in the glucagon group compared with the vehicle group, but this failed to reach statistical significance (*p* = 0.0571). There were also greater relative mRNA concentrations (Figure 3C) of genes involved in AA catabolism and ureagenesis, including PPARGC1A (421% higher, *p* = 0.0020), ARG2 (1324% higher, *p* = 0.0286), HAL (278% higher, *p* = 0.0124), and GLDC (197% higher, *p* = 0.0182), in fetal livers collected under glucagon infusion conditions compared with vehicle. The urea acid cycle and AA catabolic enzymes ARG1, GLS1, GLS2, BCAT1, and BCAT2 were not different between groups.

**Figure 4:**
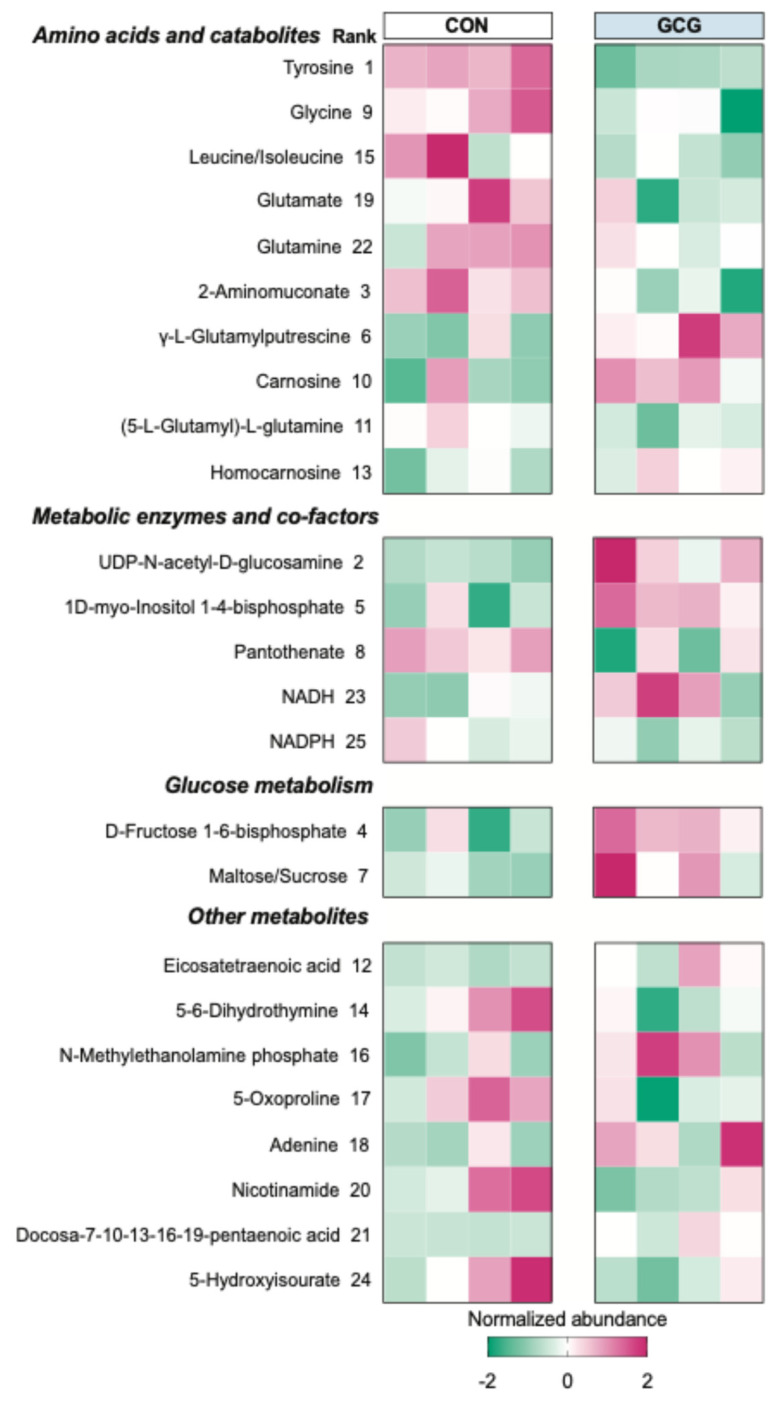
Two-hour fetal glucagon infusion increases metabolites associated with amino acid catabolism in the liver. The top 25 metabolites as determined by variable importance in projection (VIP).

**Figure 5:**
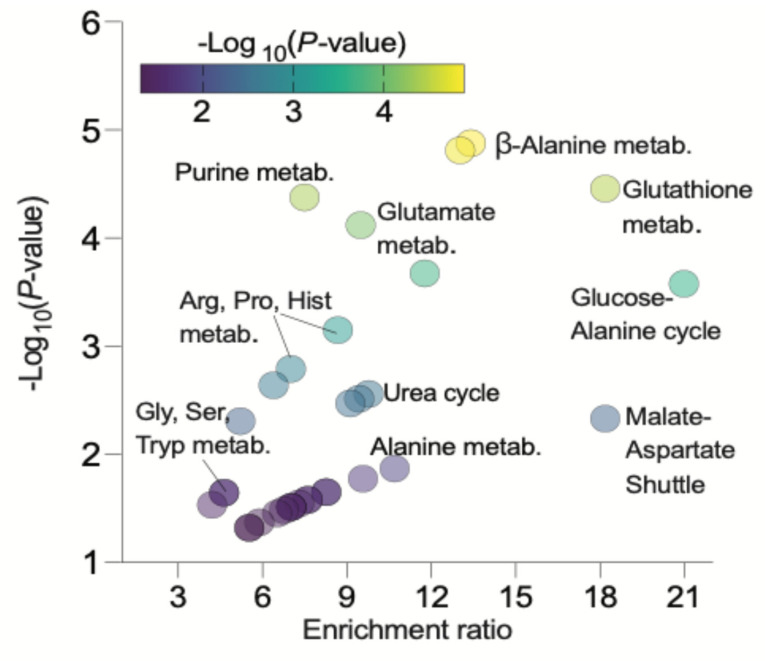
Bubble plot of enriched pathways identified after two-hour fetal glucagon infusions based on top 25 VIP metabolites. Log*p* values ≤ 1.30 were used as a threshold for identifying significantly enriched pathways, and enrichment scores were calculated as a ratio of total hits to expected hits. Rank is determined by VIP.

To better understand the impact of 2 h fetal glucagon infusions on hepatic metabolism, we conducted semi-targeted metabolomics. We employed a multivariate PLS-DA to differentiate between vehicle control and glucagon-treated samples to identify metabolites regulated by glucagon. The top 25 metabolites with the highest variable projection (VIP) scores are highlighted in Figure 4. Notably, metabolites regulated by acute hepatic exposure to glucagon included amino acids such as tyrosine, glycine, leucine/isoleucine, glutamate, and glutamine, along with AA catabolites: 2-aminomuconate, γ-L-glutamylputrescine, carnosine, 5-L-glutayl-L-glutamine, and homocarnosine. Glucagon also decreased NADH and increased NADPH, as well as elevated metabolites and enzymes linked to glucose metabolism. Subsequently, we performed pathway enrichment analysis on the top 25 VIP metabolites (Figure 5). Enriched pathways following acute glucagon infusion included beta-alanine metabolism, NAD+ metabolism, glutathione metabolism, purine metabolism, glutamate metabolism, ammonia recycling, the glucose-alanine cycle, histidine metabolism, arginine and proline metabolism, the urea cycle, lysine degradation, the malate-aspartate shuttle, and tyrosine metabolism.

### 2.5. Isolated Fetal Hepatocyte Studies

**Figure 6:**
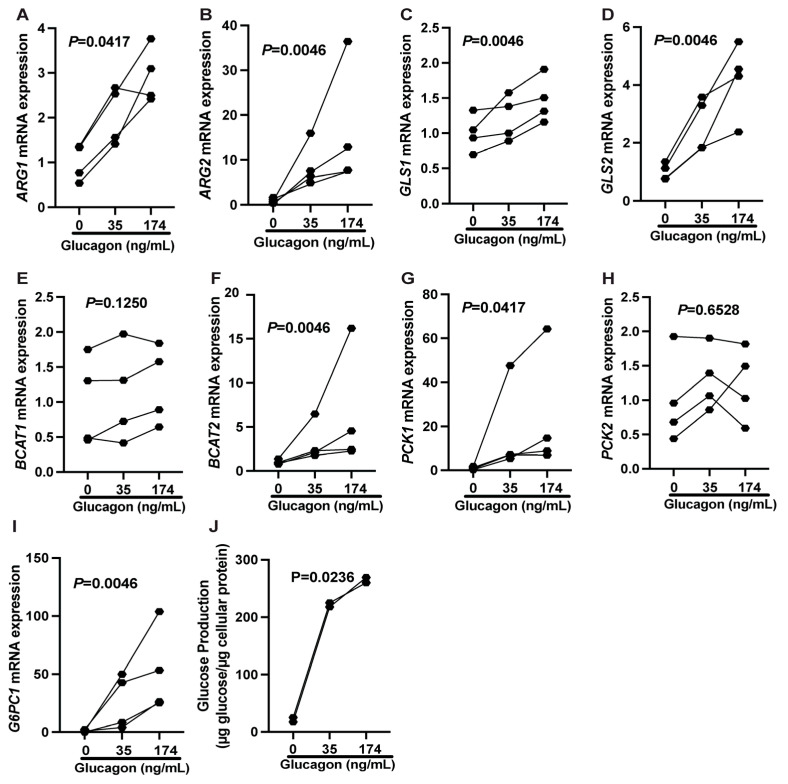
Twenty-four-hour glucagon treatment increases mRNA concentrations of several genes related to glucose and amino acid as well as glucose production in isolated fetal sheep hepatocytes. (**A**–**I**) Isolated fetal hepatocytes from non-infused pregnancies were treated with 0, 32, or 160 ng/mL of glucagon for 24 h. mRNA concentrations were normalized to the average of two reference genes and presented as fold change from the 0 ng/mL condition. (**J**) Fetal hepatic glucose increased in a dose-dependent manner in isolated hepatocytes in response to glucagon. Symbols represent individual hepatocyte preparations. Results were compared using a nonparametric repeated measures ANOVA. Individual values and *p*-Values are shown.

To separate the hepatic stimulatory actions of glucagon from potential in vivo cofounders, isolated hepatocytes were collected from additional control sheep pregnancies and cultured in conditions without insulin at increasing concentrations of glucagon (0 ng/mL, 35 ng/mL, and 174 ng/mL) to directly test the impacts of glucagon on hepatocytes. Glucagon treatment increased relative mRNA concentrations (Figure 6A–I) of both AA catabolizing ARG1 (*p* = 0.0417), ARG2 (*p* = 0.0046), GLS1 (*p* = 0.0046), GLS2 (*p* = 0.0046), BCAT2 (*p* = 0.0046), PPARGC1A (*p* = 0.0046), and gluconeogenic PCK1 (*p* = 0.0417) and G6PC1 (*p* = 0.0046) genes. Likewise, glucagon increased glucose production (*p* = 0.0236, Figure 6J).

## 3. Discussion

This study aimed to test the hypothesis that a two-hour fetal glucagon infusion would lower fetal AA concentrations by both stimulating hepatic AA catabolism and inhibiting placental AA transfer. Our results demonstrate that while a two-hour fetal glucagon infusion had minimal impact on the placenta, it did stimulate hepatic AA catabolism by increasing several AA catabolic enzymes and regulators, which resulted in reduced concentrations of most fetal AAs. Our data also suggest that glucagon stimulates gluconeogenesis in the fetal liver due to elevated gluconeogenic enzymes, increasing hepatic glucose production.

The physiological role of glucagon in regulating fetal AA concentrations is supported by several studies. Teng et al. [16] identified glucagon as a contributor to the regulation of fetal AA concentrations, along with somatostatin [16]. Furthermore, recent evidence from Cilvik et al. [17] observed acute drops in fetal AA concentrations 24 h into fetal glucagon infusions, along with a concurrent 3-fold increase in fetal insulin concentrations which is similar to what was observed in the current study. Combined with our previous work demonstrating increased fetal glucagon concentrations in response to elevated AA concentrations [18,19,20,21], our findings support the crosstalk between the pancreatic α-cell and liver, which is activated during acute periods of elevated glucagon, similar to the adult [6,7].

### 3.1. Glucagon and the Liver

The stimulatory effects of glucagon on hepatic AA catabolism in the fetus are supported by the robust transcriptional increase in several key genes in the present study. This includes arginase isoform 2 (ARG2). ARG2 is the rate-limiting enzyme and the final step in the AA catabolic urea cycle pathway. This increase is consistent with a previous report that exogenous fetal administration of glucagon in the rat also increased arginase activity in vitro from the term fetal liver [22]. However, this previous in vitro work suggested that the ARG2 increase was dependent on a glucocorticoid–glucagon interaction, while the present study demonstrates that glucagon alone is sufficient to stimulate this response in fetal sheep hepatocytes. In addition to ARG2, glucagon increased relative peroxisome proliferator-activated receptor gamma co-activator 1-alpha (PPARGC1A) mRNA concentrations. PPARGC1A is a co-activator in the liver that promotes urea production via the urea cycle [23]. This relationship has been previously demonstrated in vitro using murine (C57BL/6) primary hepatocytes incubated with glucagon [24]. Furthermore, we found that glucagon increased histidine ammonia-lyase (HAL) and glycine decarboxylase (GLDC) mRNA. HAL catalyzes the first deamination step of histidine catabolism, while GLDC is the rate-limiting enzyme for glycine catabolism. Glucagon has been shown to increase both HAL and GLDC in adult rodent studies [25]. GLDC is the rate-limiting enzyme for glycine catabolism and a mechanism for maintaining glycine homeostasis and has previously been demonstrated to be increased by glucagon in adult mice [26]. The observation in the current study that glucagon increases hepatic mRNA concentrations of both HAL and GLDC is consistent with lower fetal plasma histidine and glycine concentrations. This is further supported by the metabolomic analysis of fetal liver tissue, which revealed increased catabolism of numerous Aas, including beta-alanine, glutamate, histidine, arginine, proline, lysine, and tyrosine after 2 h acute fetal glucagon infusion. Furthermore, those livers also had greater NADH concentrations and lower NADPH concentrations, combined with greater ammonia recycling and urea cycle enzymes. Altogether, these data support the idea that an increase in hepatic AA catabolism is largely responsible for the reduced fetal AA concentrations. The current study expands the findings of glucagon-stimulated hepatic AA catabolism to a different species (sheep) at a different developmental time point (late gestation fetus).

Our observations regarding glucagon potentiation of AA catabolism are further supported in vitro in cultured hepatocytes. To fully capture any possible effect of glucagon, we selected a longer duration (24 h) and higher concentrations of glucagon (35 and 174 ng/mL). mRNA relative concentrations of both arginases and PPARGC1A were elevated, along with both glutaminases (GLS1 and GLS2) and branched-chain amino acid transferase 2 (BCAT2) in hepatocytes exposed to glucagon. The greater increases in the AA catabolic and gluconeogenic genes in vitro cultured hepatocytes could be partially explained by the longer glucagon exposure time (24 h vs. two-hour) but still support the stimulatory role of glucagon on hepatic metabolism.

To determine if glucagon may have direct effects on the fetal liver, we histologically examined the fetal liver’s GCGR mRNA and protein. As expected, there was widespread expression of GCGR on hepatocytes present throughout the fetal liver. Acute glucagon exposure caused a redistribution of the GCGR, as evidenced by IHC, which could possibly indicate internalization. This response is well documented in hepatic tissue in acute response to the ligand [27,28].

In addition to the physiological increase in fetal glucose concentrations, we also found higher relative liver mRNA concentrations of glucose-6-phosphatase (G6PC1) and of both phosphoenolpyruvate carboxykinase isoforms. These mRNAs encode enzymes that catalyze the rate-limiting (PCK1 and PCK2) and final committed steps (G6PC1) of gluconeogenesis. Elevated PCK1 and PCK2 are seen in other conditions, such as FGR [10,29], that result in elevated glucagon concentrations. Moreover, we also demonstrated a significant reduction in hepatic glycogen at the end of the glucagon infusion and a dose-dependent increase in hepatic glucose production in response to glucagon in the primary culture of fetal ovine hepatocytes. Together, these data indicate that hepatic glucose production via gluconeogenesis and glycogenolysis are responsible for the acute increase in fetal glucose concentrations. In adults, the action of glucagon on hepatic AA metabolism favors the catabolism of gluconeogenic AAs to provide substrates for gluconeogenesis [30]. The same phenomena are likely occurring in the current study observed in the fetus.

### 3.2. Glucagon and the Placenta

In addition to glucagon’s effects on hepatic AA catabolism, we also hypothesized that an acute fetal glucagon infusion would inhibit placental AA transport. Our previous study [15] investigating the effects of chronic (8–10 d) late gestation fetal hyperglucagonemia demonstrated lower placental AA transport into fetal circulation, as well as lower uterine blood flow, and lower uterine uptake of nutrients (AAs, oxygen, glucose). In contrast, the acute glucagon infusion tested in the present study failed to impact the maternal–fetal nutrient transport or blood flows. Therefore, the acute decrease in fetal AA concentrations in the current study is likely due to higher hepatic AA catabolism rather than lower placental AA transport.

Because we did not observe immediate changes in placental AA transport due to acute fetal glucagon infusions, we investigated glucagon receptor distribution in the sheep placenta. Few studies to date have shown glucagon receptors in placentas, but we previously identified GCGR mRNA in sheep fetal placental tissue via real-time quantitative PCR [15]. This approach was also used to identify GCGR mRNA in mouse placentas [31]. Additionally, the presence of glucagon receptors in the human placenta has been based on glucagon’s biological effects, although the receptor itself was not directly examined [32]. Supporting these earlier results, both the mRNA and protein product of the GCGR were identified in situ within the trophoblast cells of the sheep placenta but do not appear to internalize due to glucagon exposure. This suggests the lack of change in placental AA uptake in the current study is not simply due to a lack of receptors but requires longer exposure to elevated fetal glucagon concentrations compared with the fetal liver.

The only change in maternal–fetal nutrient transport that we identified was a reduction in the amount of glucose transported by the placenta into the fetal circulation (umbilical glucose uptake). Glucose is transported from the maternal to fetal circulation by the placenta via transporter-mediated facilitated diffusion [33,34], which is dependent on a maternal-to-fetal glucose concentration gradient. When the transplacental glucose gradient decreases, such as during an increase in fetal glucose concentrations without a change in maternal glucose concentrations, placental glucose transport will decrease [35]. The decrease in umbilical uptake has been reported previously for acute glucagon infusions of this dose [36]. We speculate that the increase in fetal glucose concentrations in vivo is secondary to a glucagon-stimulated increase in fetal hepatic glucose production.

Our previous research has suggested a link between fetal glucagon and placental function via the regulation of the placental hormone chorionic somatomammotropin (CSH). In chronic fetal hyperglucagonemia, CSH mRNA levels dropped by 40%, and secretion into maternal circulation decreased by 80% after prolonged glucagon infusion [15]. Reducing CSH via RNA interference similarly decreased uterine blood flow and nutrient transfer, resembling effects seen in hyperglucagonemia [37,38,39]. This regulatory effect of glucagon on CSH secretion is observed across species, including a dose-dependent CSH decrease in mixed populations of human cytotrophoblast and syncytiotrophoblast cells [32]. In contrast, the current study’s short-term infusions did not alter CSH expression or secretion, indicating that acute hyperglucagonemia is insufficient to impact CSH and downstream placental function. Thus, we speculate that the involvement of the placenta regulating AA is only present in a chronic setting, where it reduces placental CSH secretion, impairing uterine blood flow and nutrient transfer, rather than through immediate effects on nutrient uptake [15]. Further studies are required to test this relationship and understand at what stage placental AA transfer is reduced.

### 3.3. Proposed Model of Acute Glucagon Action in the Fetus

**Figure 7:**
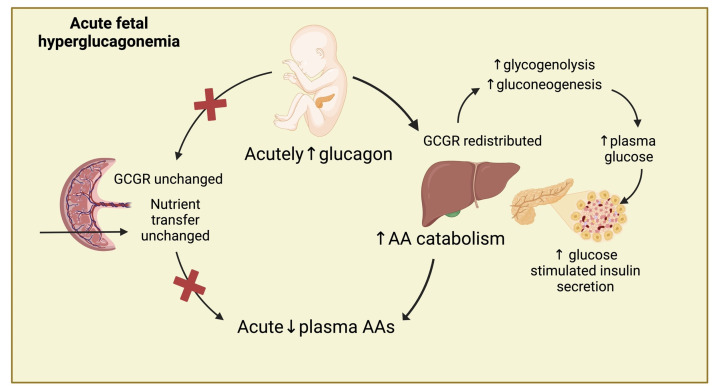
During periods of acutely elevated fetal glucagon, circulating AA concentrations are lowered by glucagon stimulation of hepatic AA catabolism. Glucagon also causes a redistribution of glucagon receptors in the fetal liver and stimulates hepatic glucose production through both glycogenolysis and gluconeogenesis. This elevates fetal glucose concentrations, resulting in glucose-stimulated insulin secretion. In contrast to the acute effects in the liver and previously published chronic effects on the placenta, acute fetal hyperglucagonemia has minimal impact on placental nutrient transport. Created with BioRender.com.

We speculate that the AA-glucagon relationship in the fetus is more complex than in the adult due to additional crosstalk from the placenta that likely regulates fetal AA concentrations, especially during extended periods of hyperglucagonemia. With short-duration (two-hour) glucagon exposure (Figure 7), our data support that hepatic regulation of AA, not placental, is contributing to fetal AA concentrations, similar to the adult, and this response likely involves the AA-glucagon feedback loop. In addition, we show that glucagon induces redistribution of glucagon receptors in the fetal liver, followed by the stimulation of hepatic AA catabolizing enzymes. This makes the fetal liver the primary target for glucagon’s actions in stimulating AA catabolism in the acute setting. Additionally, in the acute setting, our data support active hepatic glucose production through both glycogenolysis and gluconeogenesis, which elevates fetal glucose concentrations, resulting in an increase glucose-stimulated insulin secretion and, thus, the higher insulin concentrations that we observed. This activation is also observed in vitro, supporting the direct actions of glucagon on hepatic AA catabolism.

In conclusion, the current study provides evidence that acute experimental fetal hyperglucagonemia upregulates hepatic amino-acid catabolism, which contributes to the glucagon-mediated pancreas-liver AA feedback loop by reducing fetal AA concentrations. This supports that the late-gestation fetal liver responds similarly to the adult. This study also suggests that the duration of glucagon exposure likely impacts whether the placenta is involved in the pancreatic-liver feedback loop, and potentially, this could be coordinated placental production and secretion of CSH. Combined, these data, along with our previous findings [15], emphasize that glucagon regulates complex fetal adaptations to pregnancy complications [40]. Future directions for these investigations should examine the mechanisms by which fetal glucagon regulates placental CSH secretion and trophoblast function, as well as adaptations in this regulation associated with pregnancy complications.

## 4. Materials and Methods

### 4.1. Animal Preparation

All experiments were conducted at the Perinatal Research Center, University of Colorado School of Medicine, in compliance with the Institutional Animal Care and Use Committee and the ARRIVE 2.0 guidelines [41]. This Center is accredited by the Association for Assessment and Accreditation of Laboratory Animal Care International. Studies were conducted on Columbia-Rambouillet sheep with singleton pregnancies. Animals were anesthetized at 129 ± 3 days of gestational age (dGA), and surgeries were performed to place indwelling catheters into the lower fetal abdominal aorta, femoral vein, umbilical vein, and maternal uterine vein and femoral artery, as previously described [15,37]. Before their first study, animals were allowed to recover for seven or eight days.

### 4.2. Study Design

**Figure 8:**
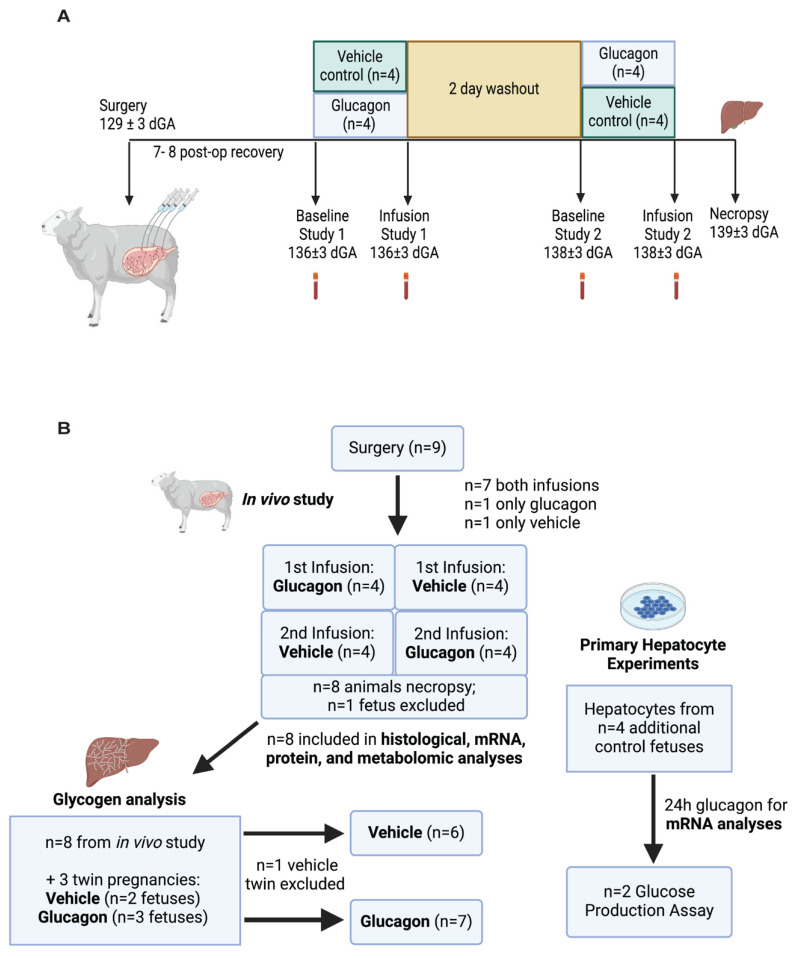
Experimental design, complications, and sample sizes. (**A**) Experimental timeline and sample size (*n*) during the two-period glucagon infusions with a crossover design with a 48 h washout period. Blood tubes represent the sampling of fetal and maternal blood as described in methods to measure blood flow and nutrient flux across the placenta. (**B**) Schematic documenting sample sizes for each set of experimental parameters. The in vivo study included a total *n* = 8. Nine animals underwent surgery, and seven had full studies, including both baseline periods and both infusions (vehicle control and glucagon). One animal underwent baseline + glucagon only, and another animal was studied under baseline and vehicle conditions only. Eight animals were sacrificed, and tissues were collected immediately following their second infusion, resulting in *n* = 4 collected under vehicle control conditions and *n* = 4 under glucagon conditions, with one fetus excluded from the necropsy. All eight samples were subjected to histological, mRNA, protein, and metabolomic analyses. All eight samples were analyzed for glycogen content, with the addition of three twin pregnancies, each with one twin receiving glucagon and the other receiving vehicle control infusions. One vehicle-control fetus was excluded from analysis for a total of *n* = 6 fetal livers collected under vehicle-control conditions and *n* = 7 collected under glucagon conditions. Both panels were created with BioRender.com.

The protocol included two study days, as shown in Figure 8A. Each study day consisted of a baseline transplacental tritiated water diffusion study, prior to any glucagon or vehicle infusion, across four blood draws to measure uterine and umbilical blood flows, uterine oxygen and nutrient uptakes, uteroplacental oxygen and nutrient utilization, and umbilical oxygen and nutrient uptakes [15,37]. This was followed by a two-hour glucagon or vehicle infusion with another transplacental tritiated water diffusion study to measure the same outcomes measured during the baseline period. The glucagon or vehicle infusion continued while blood draws were performed during this 120 min study period. Following the first two-hour glucagon or vehicle study, infusions were stopped, and animals were allowed to recover for an average of two days. After this washout period, the opposite fetal infusion and study were performed in a crossover design. During the study periods, the fetal blood removed was replaced with heparinized maternal whole blood.

The glucagon infusion (*n* = 8, performed on 136 ± 3 dGA) consisted of glucagon mixed in 0.5% (*w*/*v*) bovine serum albumin (BSA) in saline at 1.8 µg·mL^−1^. Animals were given approximately 3.6 µg of glucagon as a bolus, followed by a continuous infusion of 150 ng·min^−1^ (approximately 38 ng·kg^−1^·min^−1^) to mimic the previous infusion rate used for the chronic study [15]. The vehicle infusion (*n* = 8, performed on 136 ± 3 dGA) was 0.5% BSA in saline given at equivalent volumes and rates. A total of nine animals were studied. One animal only received a baseline and glucagon infusion study. Another animal only received a baseline and vehicle infusion study. Therefore, a total of eight animals were studied after a glucagon infusion and a total of eight animals after a vehicle infusion. The sex of the fetuses studied was four males, four females, and one unrecorded. The males and females were split evenly in terms of the order of the infusate received. In one of the animals, uterine blood flow could not be measured, which precluded calculation of uterine uptake rates and uteroplacental utilization rates during either of the studies. In a second animal, umbilical blood flow could not be measured during either of the studies. In a third animal, which only had a glucagon study and not a vehicle study, umbilical blood flow also could not be measured. Without umbilical blood flow measured we could not calculate umbilical uptake rates or uteroplacental utilization rates.

### 4.3. Biochemical Analysis

All biochemical assays have been previously described [15,37]. Briefly, whole blood was collected in heparin-coated syringes to measure hematocrit, pH, partial pressure of O_2_ (PaO_2_), partial pressure of carbon dioxide (PaCO_2_), and hemoglobin-O_2_ saturation and to calculate O_2_ content using a Blood Gas Analyzer ABL 825 (Radiometer, Copenhagen, Denmark). Whole blood was also collected in EDTA-coated syringes and centrifuged at 14,000× *g* for 3 min at 4 °C to isolate plasma for immediate measurement of glucose and lactate concentrations using a Yellow Springs Instrument 2700 (Yellow Springs Instruments, Yellow Springs, OH, USA). Aliquots of plasma were frozen at −80 °C for measurement of AAs and hormones. Plasma AA concentrations were measured using a Dionex TM ICS 5000+ high-pressure ion chromatograph with a Pickering PCX Pinnacle 120-4 channel variable wavelength detector (Thermo Electron North America, LLC, Waltham, MA, USA). Plasma insulin, IGF-1, and cortisol concentrations were measured using enzyme-linked immunosorbent assays (ALPCO Immunoassays, Salem, NH, USA, cat #80-INSOV-E01, 22-IGFHU-E01, and 11-CORHU-E01-SLV, respectively). Plasma glucagon was measured with an ELISA (ALPCO Immunoassays; cat #48-GLUHU-E01) with inter- and intra-assay coefficients of variation 2.1% and 3.4%, respectively in accordance with Tanner et al. [39]. Plasma chorionic somatomammotropin (CSH) concentrations were assessed by radioimmunoassay as previously described [15,39,42].

### 4.4. Calculations

All calculations were previously published in detail [15,38]. Uterine and umbilical blood flow rates were measured using the transplacental tritiated water diffusion technique. Uterine and umbilical plasma flows were calculated by multiplying uterine and umbilical blood flow, respectively, by 1 minus hematocrit. Net uterine and umbilical uptake rates of oxygen, glucose, lactate, and AAs were calculated by subtracting the mean uterine venous concentration from the mean maternal arterial concentration for uterine or the mean fetal arterial concentration from the mean umbilical vein concentration for umbilical uptake of the four steady-state blood draws and multiplying those differences by uterine or umbilical plasma flow for glucose, lactate, and AAs or by uterine or umbilical blood flow for oxygen. Uteroplacental utilization rates are calculated as the difference between uterine and umbilical uptakes.

### 4.5. Placentome and Fetal Liver Collection

With the second experimental infusion (glucagon, *n* = 4, or vehicle, *n* = 4) continuing, sheep received diazepam (0.2 mg·kg^−1^) and ketamine (20 mg·kg^−1^) intravenously, and fetuses were delivered via maternal laparotomy and hysterotomy. The fetal liver was exposed, and a biopsy was taken and immediately frozen in liquid nitrogen before transfer to −80 °C. Another biopsy was taken and immediately placed in 4% neutral buffered paraformaldehyde (PFA). Following the procedure, the mother and fetus were euthanized with pentobarbital sodium (390 mg·mL^−1^ intravenously; 12 and 2 mL, respectively; Bortech Pharmaceuticals, Dearborn, MI, USA). Three randomly selected A and B type placentomes [43] were dissected from the endometrium, rinsed in ice-cold sterile saline, and dissected into maternal (caruncle) and fetal (cotyledon) components, snap frozen in liquid nitrogen, and stored at −80 °C. Another three randomly selected A and B type placentomes were immediately placed into 4% PFA.

### 4.6. Histology of the Placenta and Fetal Liver

#### 4.6.1. Immunohistochemistry

For immunohistochemical (IHC) detection of glucagon receptor (GCGR) protein, paraffin-embedded tissues were sectioned at 5 μm, put on a glass slide, baked for 1 h at 65 °C, and dehydrated with xylene and series of ethanol/water, followed by antigen retrieval using citrate buffer. Slides were immunolabeled with rabbit polyclonal anti-glucagon receptor antibody (cat #ABS551, EMD Millipore, Burlington, MA, USA) at a 1:1000 dilution. Detection was performed with an ImmPress HRP anti-Rb IgG peroxidase polymer detection kit (Vector Laboratories, Burlingame, CA, USA), followed by DAB visualization with an ImmPACT DAB peroxidase substrate kit (Vector Labs, Newark, CA, USA). Stained tissue sections were imaged using an Aperio CS2 whole slide scanner with a 40× objective (Leica Biosystems, Buffalo Grove, IL, USA). Images were qualitatively assessed using ImageScope software version 12.4.3.5008 (Leica Biosystems).

#### 4.6.2. In Situ mRNA Hybridization

Chromogenic RNAScope detection was used to perform in situ mRNA hybridization according to the manufacturer’s protocol (Advanced Cell Diagnostics, Hayward, CA, USA). Slides with 5 μm sections were deparaffinized in xylene, followed by rehydration in a series of ethanol washes. Following citrate buffer (Advanced Cell Diagnostics) antigen retrieval, slides were rinsed in deionized water and immediately treated with protease plus (Advanced Cell Diagnostics) at 40 °C for 30 min in a HybEZII hybridization oven (Advanced Cell Diagnostics). A probe directed against ovine GCGR was applied at 40 °C in the following order: target probes, preamplifier, amplifier, and label probe. After each hybridization step, slides were washed two times at room temperature. mRNA chromogenic detection was performed, followed by counterstaining with hematoxylin Mayer’s (Sigma-Aldrich, St. Louis, MO, USA). For combined mRNA and IHC staining, slides were deparaffinized and treated with protease plus following antigen retrieval; after probe against GCGR mRNA hybridization, slides were stained for GCGR protein using standard IHC protocol and finished with mRNA probe detection using standard Advanced Cell Diagnostics protocol described above. Stained tissue sections were imaged using an Aperio CS2 whole slide scanner with a 40× objective (Leica Biosystems, Buffalo Grove, IL, USA). Images were qualitatively assessed in ImageScope software (Leica Biosystems).

### 4.7. mRNA Analysis of the Placenta and Fetal Liver

RNA was extracted from approximately 200 mg of pulverized tissue as previously described [15,19]. RNA concentration was measured using a Nanodrop spectrophotometer (Thermo Fisher Scientific, Waltham, MA, USA). RNA integrity was determined on an Agilent 2100 Bioanalyzer (Agilent Technologies, Santa Clara, CA, USA). The average RNA Integrity Number was 9.3 ± 0.6, with all samples being in the acceptable range. Total RNA (2 µg) was reverse transcribed using the SuperScript III First-Strand Synthesis SuperMix (Thermo Fisher Scientific; cat #18080400), and cDNA was diluted 1:10 with sterile water. Quantitative PCR assays for chorionic somatomammotropin (*CSH*; aka placental lactogen), the glucagon receptor (*GCGR*), phosphoenolpyruvate carboxykinase-1 (*PCK1*), phosphoenolpyruvate carboxykinase-2 (*PCK2*), glucose-6-phosphatase (*G6PC1*), peroxisome proliferator-activated receptor gamma co-activator-1 alpha (*PPARGC1A*), arginase-1 (*ARG1*), arginase-2 (*ARG2*), glutaminase-1 (*GLS1*), glutaminase-2 (*GLS2*), branched-chain amino acid transaminase-1 (*BCAT1*), branched-chain amino acid transaminase-1 (*BCAT2*), histidine ammonia-lyase (*HAL*), and glycine decarboxylase (*GLDC*) were performed utilizing primers developed for sheep sequences, which are summarized in Supplemental Table S1, as previously described [12,15,19,20]. A melt-curve analysis was examined to ensure a single peak. Samples were analyzed in triplicate, and the standard curve method of relative quantification was used [11,44]. Genes of interest were normalized to the mean of two reference genes (*RPS15* and *RPS37A*), and then fold-change relative to control samples was calculated. The quantitative real-time qPCR experiments and analysis were performed according to the Minimum Information for Publication of Quantitative Real-Time PCR Experiments guidelines [45].

### 4.8. Protein Analysis of the Placenta and Fetal Liver

Protein lysates were prepared from fetal liver and cotyledonary tissue, and Western immunoblotting was performed as previously described [12]. Briefly, 30 μg of protein was electrophoresed through a 4–12% NuPAGE Bis-Tris gel (Invitrogen; Waltham, MA, USA) and transferred to a 0.20 μm pore nitrocellulose membrane. To visualize GCGR, the blot was incubated in a 1:1000 in 5% BSA in (in 5% Non-Fat Dry Milk/1× Tris-Bis Solution+ 1% Tween) of rabbit anti-GCGR (Invitrogen; cat #711633) overnight at 4 °C. After washing, the membrane was incubated with a 1:5000 dilution (in 5% Non-Fat Dry Milk/1× Tris-Bis Solution+ 1% Tween) of goat anti-rabbit (LI-COR Biosciences, Lincoln, NE, USA; cat #926-32211) for 2 h at room temperature. The equality of sample loading was measured using the Revert 700 Total Protein Stain (LI-COR, cat #926-11011). Protein bands were visualized on Odyssey Fc (LI-COR Biosciences) at 800 nM for 10 min and quantified with Image Studio 6.0 (LI-COR Biosciences). Antibody specificity was verified by the presence of a single band at the expected molecular weight. Results were quantified and expressed as a ratio of glucagon receptor (*GCGR*) signal to total protein.

### 4.9. Metabolomic Analysis of the Fetal Liver

Fetal liver tissue samples from fetuses were collected after either 2 h glucagon infusion (*n* = 4) or vehicle control (*n* = 4) conditions and subjected to targeted metabolomic profiling at the University of Colorado Metabolomic Core [46,47]. Briefly, as summarized by Wesolowski et al. [46], liver tissue (25 mg) samples were extracted in ice-cold lysis/extraction buffer (methanol/acetonitrile/water 5:3:2). Analyses were performed using a Vanquish UHPLC system coupled online to a Q Exactive mass spectrometer (Thermo Fisher Scientific). Samples were resolved over a Kinetex C18 column (2.1 × 150 mm, 1.7 μm; Phenomenex, Torrance, CA, USA) at 25 °C using a 3 min isocratic condition of 5% acetonitrile, 95% water, and 0.1% formic acid flowing at 250 μL/min or using a 9 min gradient at 400 μL/min from 5 to 95% B (A: water/0.1% formic acid; B: acetonitrile/0.1% formic acid) [48,49]. Mass spectrometry analysis and data elaboration were performed as described by D’Alessandro et al. [49]. Metabolites were excluded if more than half the samples contained a zero value, and metabolites, where less than half the samples contained a zero value, were replaced with the half minimum value for the metabolite. Peak intensity values for liver metabolites were analyzed with MetaboAnalyst 6.0 (accessed 22/08/2024). Sample normalization was performed using normalization by median with data autoscaling.

Multivariate principal component analysis was performed using partial least squares discriminant analysis (PLS-DA) with both GCG and VEH groups. A total of 186 metabolites were detected, and the two treatment groups were separated through PLS-DA analysis. The 25 metabolites with the highest variable importance in projection (VIP) scores (rank order) from the PLS-DA analysis were identified and inputted into a Pearson–Ward unsupervised heatmap generator in MetaboAnalyst 6.0. The lists of the top 25 VIP metabolites were also included in an enrichment analysis through MetaboAnalyst 6.0, with a hypergeometric test, utilizing KEGG IDs to identify the compounds, and were mapped to the SMPDB database. Log*p* values ≤ 1.30 were used as a threshold for identifying significantly enriched pathways, and enrichment scores were calculated as a ratio of total metabolite hits to expected hits.

### 4.10. Glycogen Measurement in the Fetal Liver

Glycogen content of the fetal liver was measured from pulverized tissue as previously described and is reported as mg of glycogen per gm of liver tissue [50]. Due to an outlier in the measured glycogen content in one of the animals sacrificed under vehicle conditions, three additional pregnant ewes carrying twins were used to collect fetal livers for glycogen analysis, as described in Figure 8B. These three animals underwent surgery to place vascular catheters as described above, though no uterine vein catheter was placed. Only one of the twins in each pregnancy was instrumented (two male fetuses and one female fetus). For each animal, the instrumented twin received a glucagon infusion of the same dose and duration as described above. Liver tissue was also collected from the non-instrumented twins and grouped with the fetuses that were sacrificed under vehicle conditions, though we were unable to collect liver tissue from one of the non-instrumented twins. The two non-instrumented fetuses from which liver tissue were collected were both female. The resulted in an analysis of seven fetal livers collected under glucagon clamp conditions. These were compared to six livers collected under control conditions.

### 4.11. Primary Fetal Hepatocytes

Primary fetal hepatocytes were isolated from four additional non-infused fetuses, one male and three females, with an average gestational age of 134 ± 2 days and an average fetal weight of 3242 ± 683 g, as described in Figure 8B. Hepatocytes were isolated, as previously reported [51,52]. Briefly, a piece of the right lobe of the fetal liver was flushed with perfusion buffer and then with digestion buffer containing collagenase. The digested tissue was filtered and spun, and isolated hepatocytes were washed, plated, and allowed to attach for 4 h in DMEM (1.1 mM glucose, 2 mM lactate, 2 mM glutamine, 1 mM pyruvate, 1X non-essential AAs, 1X penicillin-streptomycin) supplemented with 0.1 nM insulin, 10 mM dexamethasone, and 10% FBS. After attachment, the cells were washed and incubated in non-supplemented DMEM.

Hepatocytes from two of the animals were subjected to a glucose production assay [51]. Hepatocytes were washed twice and incubated in phenol red and glucose-free DMEM with 10 mmol/L HEPES, 0.369% NaHCO_3_, 2 mmol/L sodium pyruvate, 20 mmol/L sodium lactate with 0 ng/mL, 35 ng/mL glucagon, or 174 ng/mL glucagon (McKesson, Irving, TX, USA; cat # 976967) for 24 h in 21% oxygen and 5% carbon dioxide. Glucose in the media (glucose oxidase assay) and protein content of cells (bicinchoninic acid protein assay) were measured. Glucose released into the media is expressed relative to the protein content of the cells.

Hepatocytes from all four of the animals were used to determine the effect of glucagon (0 ng/mL, 35 ng/mL, or 174 ng/mL) on the relative concentration of specific hepatic mRNAs [53,54]. Hepatocytes isolated from each animal were exposed to all treatment levels of glucagon in triplicate. Cells were incubated overnight in the above conditions for 24 h in 21% oxygen and 5% carbon dioxide. Triplicates were pooled for each animal before RNA was extracted from the hepatocytes, reverse transcribed, and quantitative PCR performed for *PCK1*, *PCK2*, *G6PC1*, *PPARGC1A*, *ARG1*, *ARG2*, *GLS1*, *GLS2*, *BCAT1*, and *BCAT2*. mRNA of interest was normalized to the relative concentration of the reference mRNA *RPS15*, and then fold-change relative to samples with no glucagon added was calculated.

### 4.12. Statistical Analysis

Statistical analysis was performed using GraphPad Prism (V.9.0; Boston, MA, USA). Results are expressed as mean ± Standard Deviation (SD). Differences among the periods (first [baseline] or second [glucagon or vehicle]) and infusates (glucagon or vehicle) were determined with a mixed models ANOVA that included terms for the period (first or second), infusate (glucagon or vehicle), and the interaction between period and infusate. A random animal term was included to account for repeated measurements within the same animal. If the interaction effect was *p* < 0.10, then individual means were compared using Fisher’s protected least squares difference test. The relationship between pre-infusion fetal glucagon and AA concentrations was measured using simple linear regression analysis. Cotyledonary and liver relative mRNA concentration and liver glycogen content were compared using a Student’s *t*-test and the Mann–Whitney test for parametric and nonparametric data, respectively. Welches correction was used when variances between the samples were not equal. Hepatocyte relative mRNA concentrations were compared using a nonparametric repeated measures ANOVA due to non-normal distribution. *p*-Values of <0.05 were accepted as significant. Statistical methods and thresholds for metabolomic analysis were previously described.

## Figures and Tables

**Table 1:** Nutrient concentrations, uptakes, and utilizations.

	Baseline Period		Clamp Period		*p*-Values		
Variable	Veh ± SD	GGC ± SD	Veh ± SD	GCG ± SD	Treatment	Period	Treatment x Period
**Concentrations, mmol/L**							
*Uterine*							
Total amino acids	2766.9 ± 351.6	2771.3 ± 365.1	2711.6 ± 589.5	2964.3 ± 334.8	0.7457	0.1493	0.1035
Glucose	3.49 ± 0.49	3.31 ± 0.56	3.54 ± 0.60	3.56 ± 0.68	0.5634	0.1005	0.2549
Lactate	0.60 ± 0.17	0.63 ± 0.16	0.73 ± 0.27	0.71 ± 0.26	0.7835	0.2367	0.1250
Oxygen	5.45 ± 0.47	5.58 ± 0.40	5.50 ± 0.54	5.43 ± 0.45	0.6091	0.9087	0.1505
CSH, ng/mL	324.95 ± 128.99	433.47 ± 128.11	396.17 ± 236.45	498.84 ± 144.42	0.2566	0.1216	0.9447
Placental CSH secretion, ng/min	200,755 ± 176,900	34,108 ± 76,027	58,106 ± 94,427	50,147 ± 59,507	0.0855	0.2052	0.1160
*Umbilical*							
Oxygen	4.79 ± 0.47	4.88 ± 0.38	4.57 ± 0.48	4.62 ± 0.34	0.9658	0.0008	0.4356
**Blood flows and rates of nutrient uptake and utilization**							
*Uterine uptake*							
Blood flow, mL/min	1822.56 ± 571.64	1888.91 ± 403.11	2148.19 ± 752.69	1908.07 ± 461.94	0.7941	0.1959	0.4048
Oxygen, mmol/min	2.02 ± 0.45	2.14 ± 0.26	2.47 ± 0.59	2.28 ± 0.65	0.6687	0.1450	0.6320
Carbon from amino acids, μmol/min	892.10 ± 294.22	747.41 ± 413.39	991.61 ± 529.07	681.41 ± 261.03	0.4126	0.7261	0.7641
Nitrogen from amino acids, μmol/min	272.34 ± 107.67	234.65 ± 134.15	308.35 ± 162.34	191.79 ± 111.47	0.3712	0.8843	0.6097
Glucose, μmol/min	426.56 ± 186.16	308.73 ± 64.52	363.13 ± 104.64	314.82 ± 71.09	0.1295	0.7638	0.2895
Lactate, μmol/min	111.50 ± 47.25	104.08 ± 24.83	119.94 ± 55.40	103.04 ± 36.25	0.9724	0.6868	0.4270
Total carbon, μmol/min	3173.75 ± 1257.00	2287.59 ± 766.54	2833.17 ± 1045.93	2261.21 ± 330.66	0.1836	0.9349	0.3296
*Umbilical uptake*							
Umbilical blood flow, mL/min	669.48 ± 164.88	736.61 ± 201.03	730.97 ± 229.76	730.44 ± 302.70	0.3494	0.8760	0.3082
Oxygen, mmol/min,	4.79 ± 0.47	1.43 ± 0.11	4.57 ± 0.48	1.47 ± 0.32	0.3183	0.5285	0.6379
Carbon from amino acids, μmol/min	618.61 ± 120.89	646.98 ± 166.23	624.06 ± 156.57	765.77 ± 307.76	0.2186	0.4331	0.4392
Nitrogen from amino acids, μmol/min	192.61 ± 42.57	203.26 ± 50.09	199.23 ± 45.68	233.28 ± 87.82	0.2218	0.4987	0.5804
Glucose, μmol/min	120.11 ± 22.05	128.34 ± 28.50	138.77 ± 48.63	82.40 ± 47.76	0.0247	0.2113	0.0152
Lactate, μmol/min	92.58 ± 46.42	114.30 ± 45.49	112.41 ± 74.29	123.51 ± 77.63	0.7075	0.5135	0.9696
Total carbon, μmol/min	1673.81 ± 261.83	1759.90 ± 187.36	1816.51 ± 415.51	1630.74 ± 498.43	0.9653	0.9146	0.2771
*Uteroplacental utilization*							
Glucose, μmol/min	76.87 ± 33.55	160.09 ± 54.67	65.44 ± 18.86	202.70 ± 61.20	0.3756	0.7290	0.2498
Lactate production, μmol/min	183.29 ± 87.09	196.31 ± 42.02	201.54 ± 100.38	193.08 ± 49.69	0.7990	0.6996	0.5265
Oxygen, mmol/min	0.91 ± 0.83	0.76 ± 0.21	1.21 ± 1.14	0.69 ± 0.44	0.7817	0.7810	0.4993

CSH, Chorionic somatomammotropin; GCG, Glucagon (*n* = 8); Veh, Vehicle (*n* = 8).

## Data Availability

The original data presented in the study are openly available in FigShare at https://doi.org/10.6084/m9.figshare.26400382.

## Supplementary Materials

The supporting information can be downloaded at https://www.mdpi.com/article/10.3390/ijms26051904/s1.

## Abbreviations

The following abbreviations are used in this manuscript:

AA	Amino acids
ARG1	Arginase-1
ARG2	Arginase-2
BCAT1	Branched-chain amino acid transaminase-1
BCAT2	Branched-chain amino acid transaminase-2
CSH	Chorionic somatomammotropin; aka placental lactogen
GLS1	Glutaminase-1
GLS2	Glutaminase-2
GCG	Glucagon
GCGR	Glucagon receptor
GLDC	Glycine decarboxylase
G6PC1	Glucose-6-phosphatase
HAL	Histidine ammonia-lyase
PCK1	Phosphoenolpyruvate carboxykinase-1
PCK2	Phosphoenolpyruvate carboxykinase-2
PPARGC1A	Peroxisome proliferator-activated receptor gamma co-activator-1 alpha
RPS15	Ribosomal Protein S15
RPS37A	Ribosomal Protein 37A
VEH	Vehicle

## References

[1] Campbell J.E., Drucker D.J. (2015). Islet α Cells and Glucagon—Critical Regulators of Energy Homeostasis. Nat. Rev. Endocrinol..

[2] Kajani S., Laker R.C., Ratkova E., Will S., Rhodes C.J. (2024). Hepatic Glucagon Action: Beyond Glucose Mobilization. Physiol. Rev..

[3] Hartl W.H., Miyoshi H., Jahoor F., Klein S., Elahi D., Wolfe R.R. (1990). Bradykinin Attenuates Glucagon-Induced Leucine Oxidation in Humans. Am. J. Physiol..

[4] Flakoll P.J., Borel M.J., Wentzel L.S., Williams P.E., Lacy D.B., Abumrad N.N. (1994). The Role of Glucagon in the Control of Protein and Amino Acid Metabolism In Vivo. Metabolism.

[5] Charlton M.R., Adey D.B., Nair K.S. (1996). Evidence for a Catabolic Role of Glucagon During an Amino Acid Load. J. Clin. Investig..

[6] Hayashi Y., Seino Y. (2018). Regulation of Amino Acid Metabolism and Alpha-Cell Proliferation by Glucagon. J. Diabetes Investig..

[7] Holst J.J., Wewer Albrechtsen N.J., Pedersen J., Knop F.K. (2017). Glucagon and Amino Acids Are Linked in a Mutual Feedback Cycle: The Liver–α-Cell Axis. Diabetes.

[8] Johnston D.I., Bloom S.R., Greene K.R., Beard R.W. (1972). Failure of the Human Placenta to Transfer Pancreatic Glucagon. Biol. Neonate.

[9] Hubinont C., Nicolini U., Fisk N.M., Tannirandorn Y., Rodeck C.H. (1991). Endocrine Pancreatic Function in Growth-Retarded Fetuses. Obstet. Gynecol..

[10] Limesand S.W., Rozance P.J., Zerbe G.O., Hutton J.C., Hay W.W. (2006). Attenuated Insulin Release and Storage in Fetal Sheep Pancreatic Islets with Intrauterine Growth Restriction. Endocrinology.

[11] Benjamin J.S., Culpepper C.B., Brown L.D., Wesolowski S.R., Jonker S.S., Davis M.A., Limesand S.W., Wilkening R.B., Hay W.W., Rozance P.J. (2017). Chronic Anemic Hypoxemia Attenuates Glucose-Stimulated Insulin Secretion in Fetal Sheep. Am. J. Physiol. Regul. Integr. Comp. Physiol..

[12] Jones A.K., Rozance P.J., Brown L.D., Goldstrohm D.A., Hay W.W., Limesand S.W., Wesolowski S.R. (2019). Sustained Hypoxemia in Late Gestation Potentiates Hepatic Gluconeogenic Gene Expression but Does Not Activate Glucose Production in the Ovine Fetus. Am. J. Physiol. Endocrinol. Metab..

[13] Sperling M.A., Christensen R.A., Ganguli S., Anand R. (1980). Adrenergic Modulation of Pancreatic Hormone Secretion in Utero: Studies in Fetal Sheep. Pediatr. Res..

[14] Schreiner R.L., Lemons J.A., Gresham E.L. (1981). Metabolic and Hormonal Response to Chronic Maternal Fasting in the Ewe. Ann. Nutr. Metab..

[15] Cilvik S.N., Wesolowski S.R., Anthony R.V., Brown L.D., Rozance P.J. (2021). Late Gestation Fetal Hyperglucagonaemia Impairs Placental Function and Results in Diminished Fetal Protein Accretion and Decreased Fetal Growth. J. Physiol..

[16] Teng C., Battaglia F.C., Meschia G., Narkewicz M.R., Wilkening R.B. (2002). Fetal Hepatic and Umbilical Uptakes of Glucogenic Substrates during a Glucagon-Somatostatin Infusion. Am. J. Physiol. Endocrinol. Metab..

[17] Cilvik S.N., Boehmer B., Wesolowski S.R., Brown L.D., Rozance P.J. (2024). Chronic Late Gestation Fetal Hyperglucagonaemia Results in Lower Insulin Secretion, Pancreatic Mass, Islet Area, and Beta- and α-Cell Proliferation. J. Physiol..

[18] Rozance P.J., Crispo M.M., Barry J.S., O’Meara M.C., Frost M.S., Hansen K.C., Hay W.W., Brown L.D. (2009). Prolonged Maternal Amino Acid Infusion in Late-Gestation Pregnant Sheep Increases Fetal Amino Acid Oxidation. Am. J. Physiol. Endocrinol. Metab..

[19] Gadhia M.M., Maliszewski A.M., O’Meara M.C., Thorn S.R., Lavezzi J.R., Limesand S.W., Hay W.W., Brown L.D., Rozance P.J. (2013). Increased Amino Acid Supply Potentiates Glucose-Stimulated Insulin Secretion but Does Not Increase β-Cell Mass in Fetal Sheep. Am. J. Physiol. Endocrinol. Metab..

[20] Brown L.D., Davis M., Wai S., Wesolowski S.R., Hay W.W., Limesand S.W., Rozance P.J. (2016). Chronically Increased Amino Acids Improve Insulin Secretion, Pancreatic Vascularity, and Islet Size in Growth-Restricted Fetal Sheep. Endocrinology.

[21] Brown L.D., Kohn J.R., Rozance P.J., Hay W.W., Wesolowski S.R. (2017). Exogenous Amino Acids Suppress Glucose Oxidation and Potentiate Hepatic Glucose Production in Late Gestation Fetal Sheep. Am. J. Physiol. Regul. Integr. Comp. Physiol..

[22] Husson A., Vaillant R. (1982). Effects of Glucocorticosteroids and Glucagon on Argininosuccinate Synthetase, Argininosuccinase, and Arginase in Fetal Rat Liver. Endocrinology.

[23] Li L., Zhang P., Bao Z., Wang T., Liu S., Huang F. (2016). PGC-1α Promotes Ureagenesis in Mouse Periportal Hepatocytes through SIRT3 and SIRT5 in Response to Glucagon. Sci. Rep..

[24] Tavares C.D.J., Aigner S., Sharabi K., Sathe S., Mutlu B., Yeo G.W., Puigserver P. (2020). Transcriptome-Wide Analysis of PGC-1α-Binding RNAs Identifies Genes Linked to Glucagon Metabolic Action. Proc. Natl. Acad. Sci. USA.

[25] Alemán G., Torres N., Bourges H., Tovar A.R. (1998). Regulation of Histidase Gene Expression by Glucagon, Hydrocortisone, and Protein-Free/High Carbohydrate Diet in the Rat. Life Sci..

[26] Jog R., Chen G., Wang J., Leff T. (2021). Hormonal Regulation of Glycine Decarboxylase and Its Relationship to Oxidative Stress. Physiol. Rep..

[27] Authier F., Desbuquois B., De Galle B. (1992). Ligand-mediated internalization of glucagon receptors in intact rat liver. Endocrinology.

[28] Merlen C., Fabrega S., Desbuquois B., Unson C.G., Authier F. (2006). Glucagon-Mediated Internalization of Serine-Phosphorylated Glucagon Receptor and Gsalpha in Rat Liver. FEBS Lett..

[29] Zhou X., Yang H., Yan Q., Ren A., Kong Z., Tang S., Han X., Tan Z., Salem A.Z.M. (2019). Evidence for Liver Energy Metabolism Programming in Offspring Subjected to Intrauterine Undernutrition during Midgestation. Nutr. Metab..

[30] Miller R.A., Birnbaum M.J. (2016). Glucagon: Acute Actions on Hepatic Metabolism. Diabetologia.

[31] Ouhilal S., Vuguin P., Cui L., Du X.Q., Gelling R.W., Reznik S.E., Russell R., Parlow A.F., Karpovsky C., Santoro N. (2012). Hypoglycemia, Hyperglucagonemia, and Fetoplacental Defects in Glucagon Receptor Knockout Mice: A Role for Glucagon Action in Pregnancy Maintenance. Am. J. Physiol. Endocrinol. Metab..

[32] Perlman R., Halabi C., Bick T., Hochberg Z. (1988). The Human Placenta as a Target Tissue for Glucagon. Biochem. Biophys. Res. Commun..

[33] Widdas W.F. (1952). Inability of Diffusion to Account for Placental Glucose Transfer in the Sheep and Consideration of the Kinetics of a Possible Carrier Transfer. J. Physiol..

[34] Hay W.W. (1991). Energy and Substrate Requirements of the Placenta and Fetus. Proc. Nutr. Soc..

[35] Hay W.W., Molina R.A., DiGiacomo J.E., Meschia G. (1990). Model of Placental Glucose Consumption and Glucose Transfer. Am. J. Physiol..

[36] Devaskar S.U., Ganguli S., Styer D., Devaskar U.P., Sperling M.A. (1984). Glucagon and Glucose Dynamics in Sheep: Evidence for Glucagon Resistance in the Fetus. Am. J. Physiol..

[37] Tanner A.R., Lynch C.S., Ali A., Winger Q.A., Rozance P.J., Anthony R.V. (2021). Impact of Chorionic Somatomammotropin RNA Interference on Uterine Blood Flow and Placental Glucose Uptake in the Absence of Intrauterine Growth Restriction. Am. J. Physiol. Regul. Integr. Comp. Physiol..

[38] Tanner A.R., Lynch C.S., Kennedy V.C., Ali A., Winger Q.A., Rozance P.J., Anthony R.V. (2021). CSH RNA Interference Reduces Global Nutrient Uptake and Umbilical Blood Flow Resulting in Intrauterine Growth Restriction. Int. J. Mol. Sci..

[39] Tanner A.R., Kennedy V.C., Lynch C.S., Winger Q.A., Anthony R.V., Rozance P.J. (2024). Increasing Maternal Glucose Concentrations Is Insufficient to Restore Placental Glucose Transfer in Chorionic Somatomammotropin RNA Interference Pregnancies. Am. J. Physiol. Endocrinol. Metab..

[40] Gatford K.L. (2021). Back-Seat Driver: The Fetus Is Not a Passive Passenger!. J. Physiol..

[41] Percie Du Sert N., Ahluwalia A., Alam S., Avey M.T., Baker M., Browne W.J., Clark A., Cuthill I.C., Dirnagl U., Emerson M. (2020). Reporting Animal Research: Explanation and Elaboration for the ARRIVE Guidelines 2.0. PLoS Biol..

[42] Kappes S.M., Warren W.C., Pratt S.L., Liang R., Anthony R.V. (1992). Quantification and Cellular Localization of Ovine Placental Lactogen Messenger Ribonucleic Acid Expression During Mid- and Late Gestation. Endocrinology.

[43] Vatnick I., Schoknecht P.A., Darrigrand R., Bell A.W. (1991). Growth and Metabolism of the Placenta after Unilateral Fetectomy in Twin Pregnant Ewes. J. Dev. Physiol..

[44] Wong M.L., Medrano J.F. (2005). Real-Time PCR for mRNA Quantitation. BioTechniques.

[45] Bustin S.A., Benes V., Garson J.A., Hellemans J., Huggett J., Kubista M., Mueller R., Nolan T., Pfaffl M.W., Shipley G.L. (2009). The MIQE guidelines: Minimum information for publication of quantitative real-time PCR experiments. Clin. Chem..

[46] Wesolowski S.R., Mulligan C.M., Janssen R.C., Baker P.R., Bergman B.C., D’Alessandro A., Nemkov T., Maclean K.N., Jiang H., Dean T.A. (2018). Switching Obese Mothers to a Healthy Diet Improves Fetal Hypoxemia, Hepatic Metabolites, and Lipotoxicity in Non-Human Primates. Mol. Metab..

[47] Nash M.J., Dobrinskikh E., Soderborg T.K., Janssen R.C., Takahashi D.L., Dean T.A., Varlamov O., Hennebold J.D., Gannon M., Aagaard K.M. (2023). Maternal Diet Alters Long-Term Innate Immune Cell Memory in Fetal and Juvenile Hematopoietic Stem and Progenitor Cells in Nonhuman Primate Offspring. Cell Rep..

[48] Nemkov T., Hansen K.C., D’Alessandro A. (2017). A Three-Minute Method for High-Throughput Quantitative Metabolomics and Quantitative Tracing Experiments of Central Carbon and Nitrogen Pathways. Rapid Commun. Mass Spectrom..

[49] D’Alessandro A., Nemkov T., Yoshida T., Bordbar A., Palsson B.O., Hansen K.C. (2017). Citrate Metabolism in Red Blood Cells Stored in Additive Solution-3. Transfusion.

[50] Limesand S.W., Rozance P.J., Smith D., Hay W.W. (2007). Increased Insulin Sensitivity and Maintenance of Glucose Utilization Rates in Fetal Sheep with Placental Insufficiency and Intrauterine Growth Restriction. Am. J. Physiol. Endocrinol. Metab..

[51] Thorn S.R., Brown L.D., Rozance P.J., Hay W.W., Friedman J.E. (2013). Increased Hepatic Glucose Production in Fetal Sheep with Intrauterine Growth Restriction Is Not Suppressed by Insulin. Diabetes.

[52] Nash M.J., Dobrinskikh E., Wang D., Pietras E.M., Janssen R.C., Friedman J.E., Wesolowski S.R. (2024). Isolating Mononuclear Cells from Fetal Bone and Liver for Metabolic, Functional, and Immunophenotypic Analyses in Nonhuman Primates. STAR Protoc..

[53] Thorn S.R., Regnault T.R., Brown L.D., Rozance P.J., Keng J., Roper M., Wilkening R.B., Hay W.W., Friedman J.E. (2009). Intrauterine Growth Restriction Increases Fetal Hepatic Gluconeogenic Capacity and Reduces Messenger Ribonucleic Acid Translation Initiation and Nutrient Sensing in Fetal Liver and Skeletal Muscle. Endocrinology.

[54] Thorn S.R., Sekar S.M., Lavezzi J.R., O’Meara M.C., Brown L.D., Hay W.W., Rozance P.J. (2012). A Physiological Increase in Insulin Suppresses Gluconeogenic Gene Activation in Fetal Sheep with Sustained Hypoglycemia. Am. J. Physiol. Regul. Integr. Comp. Physiol..

