# Supplementary figures and images for: A Two-Hour Fetal Glucagon Infusion Stimulates Hepatic Catabolism of Amino Acids in Fetal Sheep
Source: Int J Mol Sci. 2025 Feb 22;26(5):1904. doi: 10.3390/ijms26051904 (PMC11900341; doi:10.3390/ijms26051904)

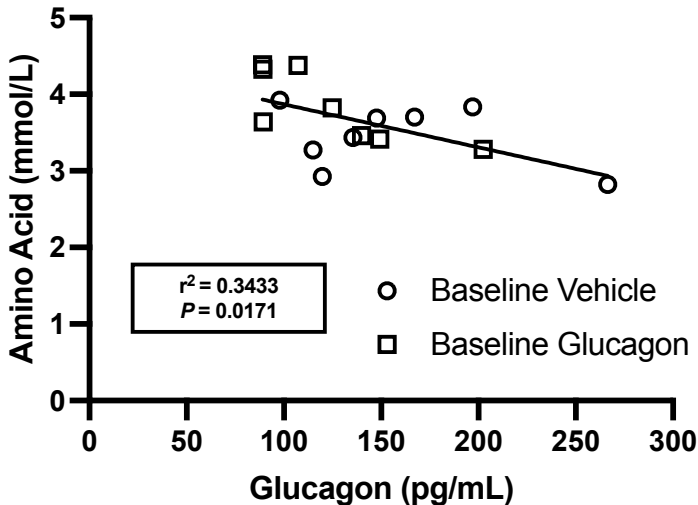

Supplement: Supplementary file 1 [file ijms-26-01904-s001.zip › Supplemental Figure S1 Relationship between fetal glucagon and amino acid concentrations.pdf]

## Cotyledon

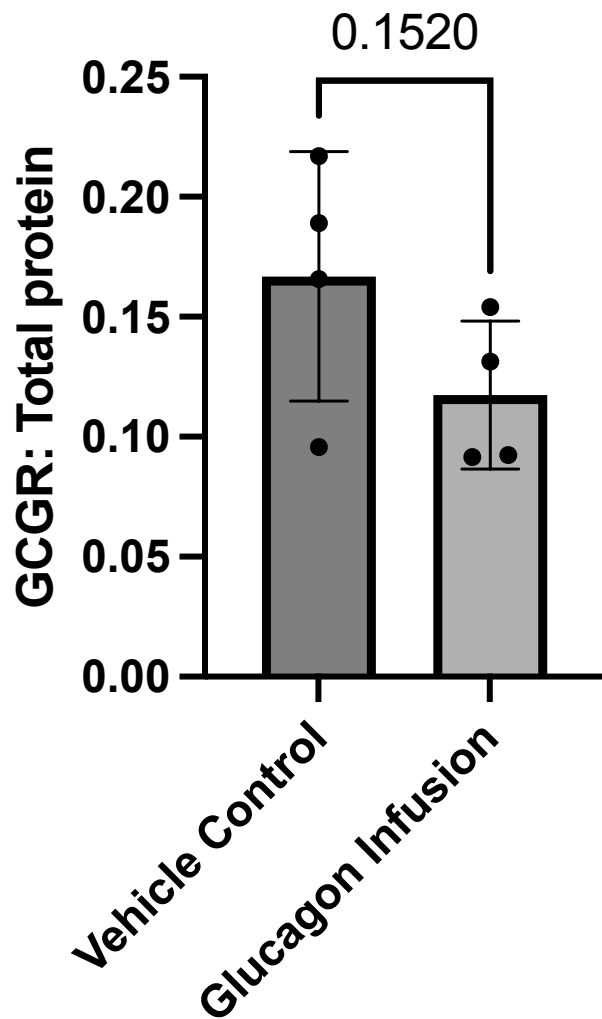

## Fetal liver

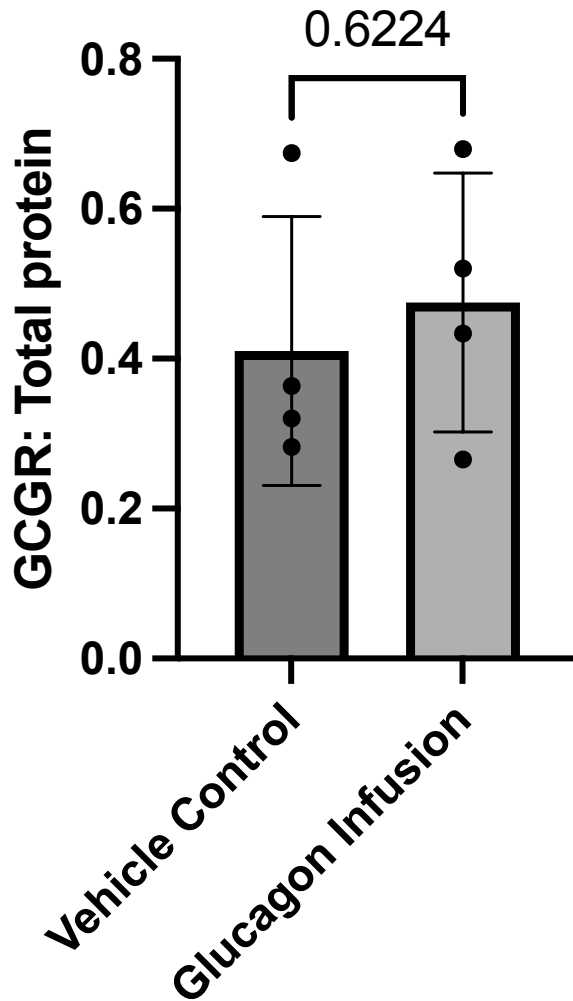

Supplement: Supplementary file 1 [file ijms-26-01904-s001.zip › Supplemental Figure S2 Glucagon receptor protein analysis of fetal liver and placenta (cotyledon).pdf]

Cotyledonary CSH mRNA  
Expression (ratio)

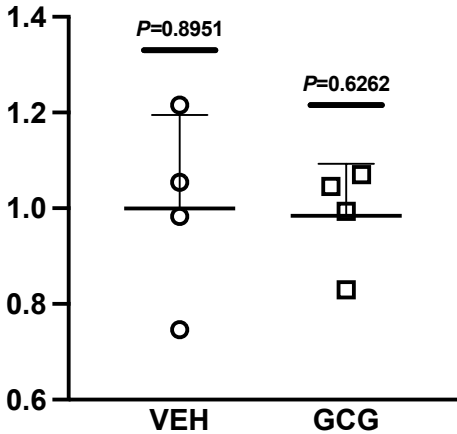

Supplement: Supplementary file 1 [file ijms-26-01904-s001.zip › Supplemental Figure S3 Placental lactogen mRNA analysis in the cotyledon.pdf]
